# Impact of cancer awareness campaigns in Peru: a 5-year Google Trends analysis

**DOI:** 10.3332/ecancer.2022.1477

**Published:** 2022-11-24

**Authors:** Jorge Luna-Abanto, Luis Gamarra, Darwin Desposorio Armestar, Benjamin Huamanchau Condori, Grivette Betsy Mendoza Tisoc, Gustavo Flores Trujillo, Elily Apumayta, Tessy Tairo-Cerrón, Cesar Centurión-Rodríguez, Luis García Ruiz, Jossué Espinoza-Figueroa, Karoll Tatiana Meza Garcia, Jorge Navarro Yovera, Milward Ubillús Trujillo, Gustavo Sarria

**Affiliations:** 1 Instituto Nacional de Enfermedades Neoplásicas, Av. Angamos Este 2520, Lima 15000, Perú; 2 Departamento de Radioterapia, Instituto Nacional de Enfermedades Neoplásicas, Av. Angamos Este 2520, Lima 15000, Perú; 3 Universidad Peruana Cayetano Heredia, Lima 15102, Perú; 4 Universidad de San Martín de Porres, Lima 15011, Perú; 5 Servicio de Anatomía Patológica, Hospital Antonio Lorena, Cusco 08001, Perú; 6 Universidad Privada Antenor Orrego, Trujillo 13008, Perú; 7 Departamento de Medicina Nuclear, Instituto Nacional de Enfermedades Neoplásicas, Av. Angamos Este 2520, Lima 15000, Perú; 8 Departamento de Medicina Oncológica, Instituto Nacional de Enfermedades Neoplásicas, Av. Angamos Este 2520, Lima 15000, Perú; 9 Departamento de Medicina Crítica, Instituto Nacional de Enfermedades Neoplásicas, Av. Angamos Este 2520, Lima 15000, Perú; 10 Departamento de Radiodiagnóstico, Instituto Nacional de Enfermedades Neoplásicas, Av. Angamos Este 2520, Lima 15000, Perú; 11 Universidad de Huánuco, Huánuco 10001, Perú; a https://orcid.org/0000-0001-8795-6635; b https://orcid.org/0000-0001-5018-2904; c https://orcid.org/0000-0003-1829-6818; d https://orcid.org/0000-0001-6792-4433; e https://orcid.org/0000-0001-7807-1931; f https://orcid.org/0000-0002-7428-411X; g https://orcid.org/0000-0002-1828-7009; h https://orcid.org/0000-0002-4565-9875; i https://orcid.org/0000-0001-9169-1895; j https://orcid.org/0000-0003-1832-7952; k https://orcid.org/0000-0002-0761-3366; l https://orcid.org/0000-0001-7136-4264; m https://orcid.org/0000-0002-3124-1224; n https://orcid.org/0000-0002-3684-9394; o https://orcid.org/0000-0002-7459-7730

**Keywords:** cancer awareness, Google Trends, cancer trends, Peru

## Abstract

**Background:**

The aim of this research was to characterise the interest on the most frequent cancers in Peru through Google Trends, its geographic and temporal relationship with massive awareness campaigns.

**Methods:**

A temporal trends analysis for the last 5 years was carried out, comparing the Relative Search Volume (RSV) with the dates of mass cancer awareness campaigns in Peru. Google Trends application was used to evaluate the interest in the topics: breast, prostate, cervical stomach and colorectal cancer between 1 January 2016 and 31 December 2020, expressed in RSV. The annual RSV for each neoplasm was compared, as well as its annual variation using the Kruskal–Wallis test. The correlation between the RSV and the estimated incidence for each province was measured using the Spearman test.

**Results:**

The topics with the highest RSV were breast (median: 20, range: 6–100) and prostate cancer (median: 28, range: 9–48). The topic ‘breast cancer’ showed a cyclical punctual increase in October, its awareness month. Searches for cervical, stomach and colorectal cancer were smaller and did not show peaks of interest. It was observed that the RSV was variable when compared with previous years (*p* < 0.05 for all the evaluated topics). Geographically, different provincial configurations of interest were observed according to neoplasia. When correlating the RSV with the incidence by province, a non-significant positive correlation (*p* > 0.05) was found for breast, cervical and colorectal cancer.

**Conclusions:**

This study suggests a positive temporal correlation between RSV and awareness cancer campaigns in Peru specially to breast cancer and, to a lesser extent, prostate cancer. Significant variations of interest were demonstrated for each neoplasm among the evaluated years. No significant correlation was found between the incidence rate and the average RSV among Peruvian provinces.

## Background

In 2018, 17 million new cases of cancer were diagnosed and 9.6 million people died from this cause [1]. By 2040, it is projected that these rates will increase fundamentally due to the growth and ageing of the world population, especially in Latin America where this demographic process is just beginning [1–3]. Globally lung, prostate, colorectal, stomach and liver cancer are the most common among men; while breast, colorectal, lung, cervical and thyroid cancer are in women [1, 3, 4]. In Peru, 69,849 new cases of cancer were reported during 2020 and there were 34,976 deaths [5]. In Peru, the cancers with the highest incidence and mortality for men were prostate cancer, gastric cancer and non-Hodgkin’s lymphoma; on the other hand, a higher incidence of cervical, breast and stomach cancer was reported in women [6].

The most widespread mass prevention and promotion campaigns on cancer are ‘pink October’ for breast cancer and ‘blue November’ for prostate cancer [2, 7], whose aim is to raise awareness on the importance of screening, early diagnosis and treatment of cancer; however, some authors have questioned the impact of these interventions [2]. According to the ‘National Comprehensive Cancer Care Plan of Peru’, screening activities include for cervical cancer: visual inspection with acetic acid, detection of human papillomavirus (HPV) through molecular testing and cervico-vaginal cytology; for breast cancer: clinical breast examination and mammography; for colorectal cancer: detection of occult blood in faeces and lower gastrointestinal endoscopy; for prostate cancer: dosage of prostate-specific antigen and digital rectal examination; and for non-screening neoplasms (such as stomach cancer), the activities are aimed at strengthening the capacities of the first level of care for the referral of suspicious cases [8].

The Google search engine has become the first and most popular source of information, and the search volume indicator could be considered a measurable unit to evaluate the interest, and indirectly the impact of cancer awareness campaigns in a viable, fast and economical way [2, 9]. There has been an increase in Internet access in Peru, from 19.8% to 76.2% between 2012 and 2019, according to the results of the residential survey of telecommunications services [10]. In this context, this research used the Google Trends application to correlate the Relative Search Volume (RSV) with the dates of cancer awareness campaigns carried out in Peru and to infer the power of the intervention. While increasing RSV is not the ultimate goal of health awareness campaigns, there is an increasing evidence of the use of the Internet, expressed in higher RSV rates, as an important platform and tool for the dissemination and implementation of public health policies [11].

## Methods

An ecological study of temporal trends was carried out on the chosen search engine, the temporal relationship between mass cancer awareness campaigns in Peru and the RSV was evaluated to establish a probable effect. The five most common neoplasms in Peru were identified according to The Global Cancer Observatory (GLOBOCAN) 2020: breast, prostate, cervical stomach and colorectal. The Google Trends application was used to obtain the weekly RSV between 1 January 2016 and 31 December 2020. The dates of the official calendar from the Peruvian Ministry of Health were taken into account, as well as the main worldwide awareness campaigns for the selected neoplasms [12].

Google Trends is a tool used to study trends and patterns of search engine queries using Google; it expresses the absolute number of searches relative to the total number of searches in each location and at each time [13]. In this application, the number of searches for each topic relative to total searches is referred to as the query share, then it is normalised to the highest volume of searches for that term over the time period being studied; this index ranges from 0 to 100, with 100 recorded on the date that saw the highest RSV activity for that term [13]. The chosen search topics were: ‘cáncer de mama’, ‘cáncer de próstata’, ‘cáncer de cérvix’, ‘cáncer de estómago’ and ‘cáncer colorrectal’. A temporal graph was made to assess the variation of the RSV according to the search topic, independently and comparatively.

The medians of RSV were compared, and the differences among groups were analysed according to the year evaluated with the non-parametric Kruskal–Wallis test. If differences were found, the Games–Howell post hoc test was performed to identify the difference in means and its directionality. Both statistical tests were considered significant if *p* < 0.05. Heat maps were made to plot the RSV by regions using the ‘R’ program. The correlation between the RSV and the incidence rate for the neoplasms evaluated by each region was evaluated by calculating Spearman’s Rho.

For statistical purpose, we assumed that the distribution of age groups was similar between regions, and the incidence rate was calculated directly from the official reported cases found at the 2018 national situational analysis of cancer. The estimated population by sex at 30 June 2016, was obtained from the National Institute of Statistics and Informatics. This study followed the current regulations regarding the ethical management of secondary data established by the The Council for International Organizations of Medical Sciences (CIOMS) standards. In this sense, approval by the institutional ethics committee was not requested.

## Results

From 1 January 2016 to 31 December 2020, the search topics with the highest RSV were ‘breast cancer’ and ‘prostate cancer’. Only the topic ‘breast cancer’ showed a trend associated with cyclical peaks of interest in October; out of these, the interest in the other topics was lower. The median RSV for breast cancer was 20 with a range of 6–100. The RSV for the topic ‘prostate cancer’ showed a greater global interest than the topic ‘breast cancer’ with a median of 28 (range: 9–48). The trend graph for ‘prostate cancer’ was characterised by little variability and no search peaks, but a higher overall average interest.

The search topics ‘cervical cancer’, ‘stomach cancer’ and ‘colorectal cancer’ had a lower RSV, they had a maximum score below 26 points. No temporal trends were found for these topics in the study period. Median RSV values were 12 (range: 0–26), 12 (range: 0–24) and 5 (range: 0–9) for the topics ‘cervical cancer’, ‘stomach cancer’ and ‘colorectal cancer’, respectively.

**Figure 1.**
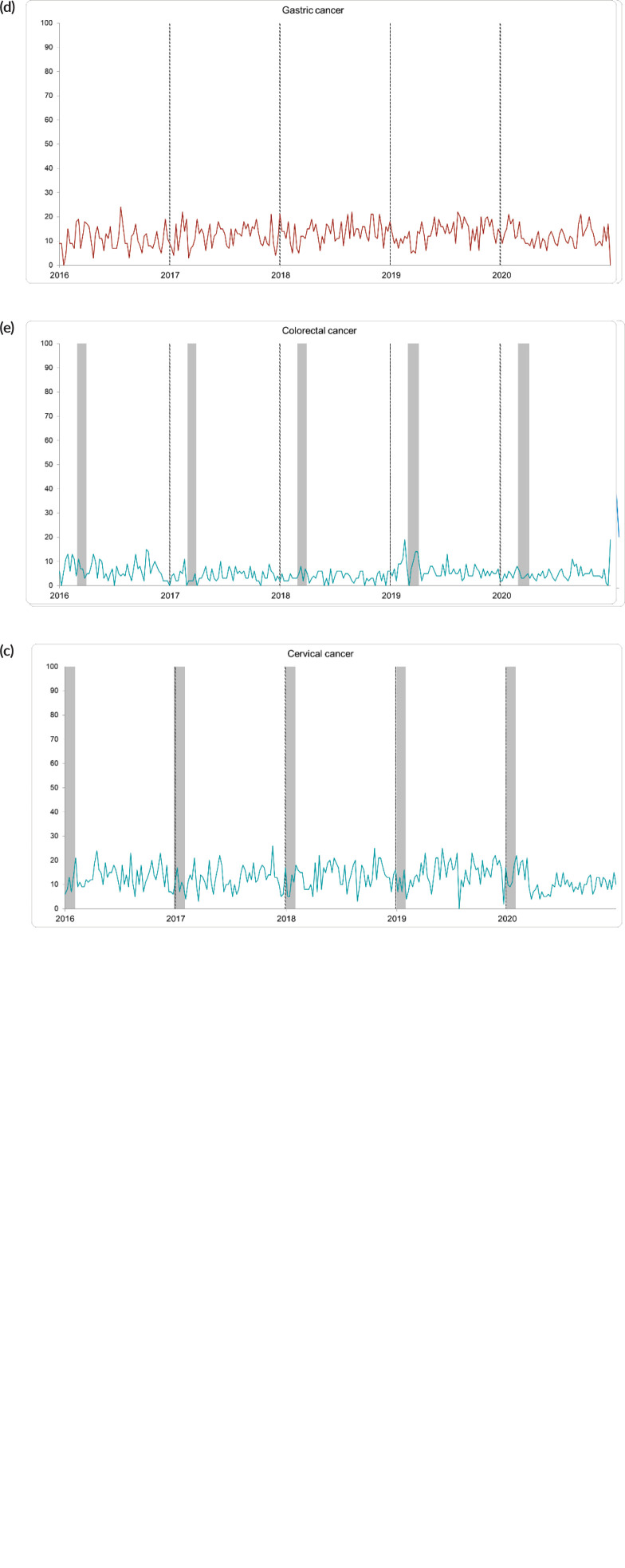
The 5-years RSV trend for (a) breast, (b) prostate, (c) cervical, (d) gastric and (e) colorectal cancer. The grey bar shows the dates of national awareness campaigns for each neoplasm.

When comparing the annual RSV between each neoplasm, a significant difference was found using the Kruskal-Wallis test. with H: 16,997 (*p*: 0.02), 15,116 (*p*: 0.04), 20,040 (*p*: 0.00), 14,552 (*p*: 0.006), 32,252 (*p*: 0.00) for ‘cancer’ subjects breast’, ‘prostate cancer”, “cervical cancer”, “stomach cancer” and “colorectal cancer”, respectively (Figure 1). When analysing these differences with the Games–Howell test, a greater interest was observed for the topic ‘breast cancer’ during 2019 compared to other years. However, this difference was only significant for the years 2016 (mean difference: 7.35; *p*: 0.018) and 2017 (mean difference: 7.74; *p*: 0.015). The interest in prostate cancer was greater during 2019 when compared to the years 2016 and 2017 with a mean difference of 4.02 (*p*: 0.034) and 4.59 (*p*: 0.012). In 2020, the interest for this neoplasm was higher than that observed in 2017 (mean difference: 3.94; *p*: 0.044).

Searches for the topic ‘cervical cancer’ decreased for 2020, when compared to the previous year’s 2016 (mean difference: −2.52; *p*: 0.042), 2017 (mean difference: −1.86; *p*: 0.230), 2018 (mean difference: −3.02; *p*: 0.014), 2019 (mean difference: −3.96; *p*: 0.001). Interest in ‘stomach cancer’ increased significantly for the years 2018 (mean difference: 2.85; *p*: 0.017) and 2019 (mean difference: 2.79 *p*: 0.023) compared to 2016. However, for 2020, a non-significant decrease in RSV was reported compared to the previous year. When analysing the RSV for colorectal cancer, a sustained decrease was observed until 2018. During 2019, an increase in interest was reported with respect to the years 2017 (mean difference: 2.23; *p*: 0.003) and 2018 (mean difference: 2.62; *p*: 0.00); no variations were found for 2020.

**Figure 2.**
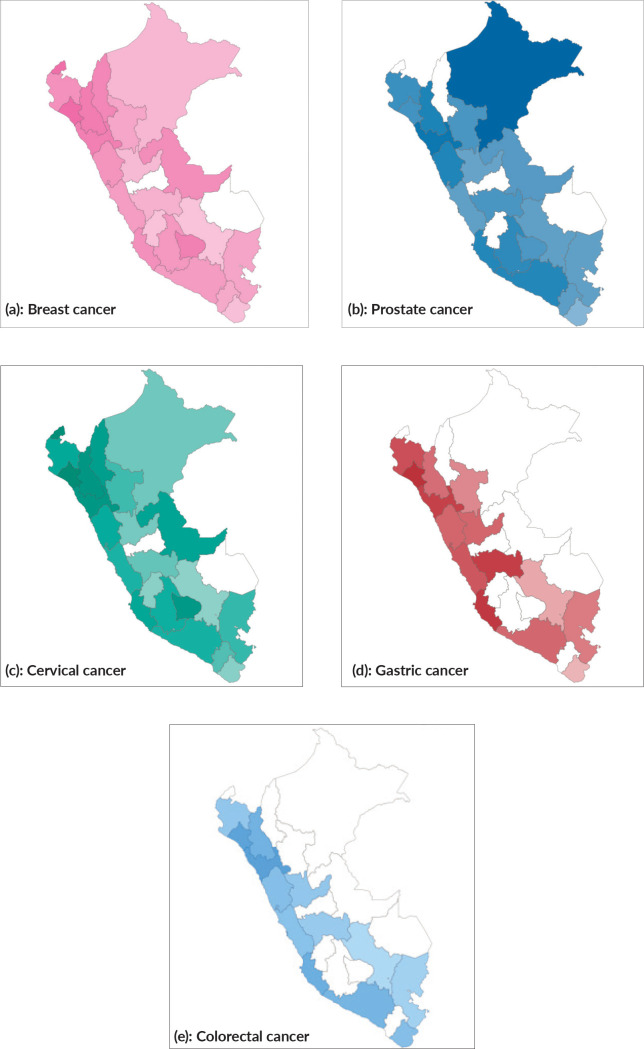
(a)– (e): Heat map of Peru, distributed by provinces according to the RSV for each topic. (a): breast cancer, (b): prostate cancer, (c): cervical cancer, (d): gastric cancer and (e): colorectal cancer.

The RSV by topic was variable depending on the geography, and various groups of provinces had greater interest in certain neoplasms. It was observed that the interest on ‘breast cancer’ was greater in the northwestern regions of the country (Lambayeque, Tumbes, Cajamarca and La Libertad). On the other hand, the provinces with less interest were located in the southeast of the country. The RSV for ‘prostate cancer’ was higher in coastal North West provinces (La Libertad, Cajamarca, Áncash); however, the province with the greatest interest in the subject was Loreto, located in the Peruvian jungle. Similarly, the distribution of interest for ‘cervical cancer’ was higher in Loreto followed by Huánuco, a central highland province, and with less interest in the coast. The distribution of interest in the topics ‘stomach cancer’ and ‘colorectal cancer’ predominated in the coastal provinces and, in the case of the former, with a certain tendency towards the central highlands. A positive correlation was found for interest in breast cancer (Rho: 0.388; *p*: 0.055), cervical (Rho: 0.098; *p*: 0.640) and colorectal (Rho: 0.386; *p*: 0.056); however, they were not significant (Figure 2).

## Discussion

### Breast cancer

The RSV for the topic ‘breast cancer’ was one of the highest and showed cyclical increases in October of each year (median: 20, range: 6–100). Similar behaviour of interest in this neoplasm was reported by Quintanilha *et al* [2] and Vasconcellos-Silva *et al* [14] who evaluated it in Brazil, same results were reported in Finland, the United States and Malaysia coinciding with October on a cyclical basis [4, 15, 16]. The most popular awareness campaign for breast cancer is ‘pink October’, which correlates with the specific increases observed in this study [17]. In Peru, the ‘Pink Day’ was established within the third week of October ‘Peru against Cancer’ campaign [18–20]. It was observed that the RSV of breast cancer increased significantly towards 2019; however, by 2020, the interest decreased probably with the increasing interest on the COVID-19 pandemic, which suggests that interest could be modified by temporary events.

The provinces of Tumbes, Lambayeque, Cajamarca, and La Libertad had the highest RSV values in this period. According to the situational analysis of cancer in Peru, the northwestern region reported the highest incidence rates for this neoplasm [21]. The correlation between RSV and the incidence of breast cancer was positive, but non-significant (Rho: 0.388; *p*: 0.055). On the other hand, a recent epidemiological study found a higher mortality rate from breast cancer in coastal provinces; this epidemiological profile could be related to the greater interest in this specific population [22] Figure 1(a) and Figure 2(a).

### Prostate cancer

The topic ‘prostate cancer’ did not show cyclical increases in RSV (median: 28, range 9–48). Khan *et al* [23] found no association between ‘Movember’ and the RSV for prostate cancer between 2004 and 2015. Patel *et al* [24] also observed a non-significant increase in interest in prostate cancer, although there was a slight increase of 4.1% in November searches. Prostate cancer prevention campaigns date back to 2003, when ‘Blue November’ and ‘Prostate Cancer Awareness Month’ were established in November and September, respectively [24]. In 2012, international recommendations shifted towards less use of prostate-specific antigen, which probably temporarily influenced the messages of awareness campaigns [25]. In contrast, a tendency to minimise the risk of prostate cancer has been reported among patients due to the absence of symptoms in the early stages of the disease, which could be related to the low RSV compared to other neoplasms [24].

Historically, in Peru, there were no mass screening campaigns and cases of advanced prostate cancer are frequent [26]; however, in recent years, November has been promoted as the ‘international month against prostate cancer’ [27, 28]. The median interest in this neoplasm was higher than that observed for breast cancer, and it increased consistently until 2019 to remain unchanged towards 2020 (*p*: 0.044). The difference with breast cancer could be related to a slow-progressing disease, an asymptomatic natural history in the early stages and influenced by other circumstantial factors [26]. The provinces with the highest RSV for prostate cancer were Loreto, La Libertad and Cajamarca. No correlation was found when comparing the interest on prostate cancer with the estimated incidence and mortality from 2014 to 2018 [21, 29] Figure 1(b) and Figure 2(b).

### Cervical cancer

The trend for cervical cancer interest remained stable and without peaks in the last 5 years, this would be related to the lack of campaigns focused on specific dates, age and cultural groups. Another hypothesis is that Peruvian awareness campaigns are scarce and with no month predilection, in congruence with public policies [30–32]. In the US, a study reported that for January, the interest on cervical cancer was significantly higher than the rest of the year (RSV: 69), in relation to the awareness month in this country [33]. On the contrary, in Peru, a sustained decrease in RSV was observed for this neoplasm in 2020 when compared to previous years. This could be related to a ‘pandemic effect’, precarious Internet access of the affected population and the lack of explicit awareness campaigns targeting the population of interest [9]. The RSV of cervical cancer was higher in the eastern and central provinces such as Loreto, Junín, Lambayeque, La Libertad and Cajamarca. The mortality rate due to this neoplasm during the period 2000–2011 was predominant in the departments of Ucayali, Loreto, Madre de Dios, Huánuco, San Martín, Amazonas, Pasco, Tacna and La Libertad with rates above the average [33]. Despite this, no significant correlation was found between the incidence rate and RSV (Rho: 0.098; *p*: 0.640). Incidence and mortality studies revealed that these rates have remained in the first places for the evaluated regions, while they decreased in metropolitan Lima [34, 35], this could be related to the access to health and Internet. Cervical cancer continues to be one of the main causes of morbidity and mortality in Peru, in this sense it would be valid to consider an awareness strategy targeting women in high prevalence areas, using digital media, which have shown to increase interest in cervical cancer knowledge and prevention [8, 36–38] Figure 1(c) and Figure 2(c).

### Gastric cancer

Although gastric cancer is one of the most frequent and deadly cancers in Peru, interest in this topic was low in the last years (median RSV: 12, range: 0–24). In this research, the interest in this neoplasm increased significantly towards 2019 (*p*: 0.017), to remain stable in 2020 despite having lower RSV than the previous neoplasms. The implementation of massive campaigns aimed at raising awareness and preventing this neoplasm is scarce and controversial, although some countries have instituted November for its promotion [39]. To date, the only country that maintains a screening and awareness programme is Japan, where gastric cancer is currently the fifth in incidence and fourth in mortality [40–42]. In the UK, a pilot oesophagogastric cancer awareness campaign was conducted; during this period, the number of referrals for endoscopy increased by 52% in the exposed geographic areas [43, 44]. But, despite having achieved the communication of an early detection message, some experts maintain that, due to the non-specificity of the symptoms associated with this neoplasm, this campaign could have led to an increase in endoscopies performed, without necessarily being associated with a higher rate of cancer detection, increased costs and waiting time [40].

Another factor that could influence the interest on gastric cancer is the low level of knowledge of the population, a Chinese study and another Peruvian study concluded that the general population has a low to medium level of awareness on the topic [45, 46]. The coastal provinces: La Libertad, Lambayeque, Sierra: Áncash, Junín and Huánuco had higher RSV for this search topic, which could be related to the fact that these regions correspond to the areas with the highest incidence and mortality from gastric cancer in recent years, a fact that has been associated with social determinants of health such as: poverty, sanitation and exposure to other risk factors [47–49]. The correlation between incidence and RSV was not statistically significant. This may be due to the cross-sectional nature of this analysis, despite the geographical relationship being clear in the heat map Figure 1(d) and Figure 2(d).

### Colorectal cancer

The interest on colorectal cancer was low (RSV: 5, range: 0–9), with no peaks of interest in the last 5 years. The main awareness campaigns on this neoplasm take place in March, other researchers have shown that these have a positive impact on the population’s interest in the neoplasm; however, this did not translate into a greater number of colonoscopies performed [50, 51]. In contrast, a Brazilian study did not find peaks of interest in the topic and a clear polarisation of interest between its regions [2]. Other American studies failed to identify cyclical peaks in relation to awareness campaigns either, this information is comparable to our findings [4, 9]. In Peru, a significant increase in RSV was observed towards 2019–2020, and its correlation with incidence rates was positive but not significant. Between 2014 and 2018, colorectal cancer was the fifth most frequent neoplasm in Lambayeque, Cajamarca and Callao; and the sixth in La Libertad and Ancash, these being the departments with the highest RSV on the subject [21]. The little interest in this neoplasm could be attributed to various factors, including its relative infrequency compared to others, a more latent clinic in its initial stages or the lack of massive campaigns. Another factor could be related to the lack of well-known Peruvians who suffer from this neoplasm, as observed with the actor Chadwick Boseman who, after his death from colorectal cancer was confirmed, an increase in interest in this neoplasm was observed among African-Americans [52] Figure 1(e) and Figure 2(e).

### Geography and internet access

Peru has three geographic regions: the coast, the highlands and the Jungle, and it is accompanied by differences in language and Internet access. According to the National Institute of Statistics and Informatics, the percentage of the population that made use of Internet between 2016 and 2020 increased 18.6% (from 57.6% to 76.2%) on the coast, 19.4% (from 31.4% to 50.8%) in the highlands and 18.3% (from 27.5% to 45.8%) in the jungle [53]. Internet access in the last 5 years has increased to a greater extent in the population with lower socioeconomic and educational levels. Thus, in urban areas, the population with elementary education (or lower) has increased its access by 17.9% (from 26.9% to 44.8%) as opposed to the population with higher university education, which increased by 6% (from 89.5% to 95.7%); and in rural areas, the population with elementary education (or lower) has increased its access by 16% (from 3% to 19%) slightly more than the population with higher education, which increased by 14.3% (from 73.5% to 87.8%) [53]. All this could explain the differences in RSV between the different Peruvian provinces.

### Limitations and implications

The main limitations of this study include the non-inclusion of other search engines and the possible existence of some systematic error in the Google Trends algorithm, that were not accessible [4]. Other limitations include poor Internet access in some provinces which are most affected by certain neoplasms, as well as the influence of events related to celebrities, seasonal influence that could be linked specially to breast cancer and the age of the users who perform searches, which were not evaluated in this research. On the other hand, the effects of the COVID-19 pandemic could be associated with a decrease of interest in neoplasms and their early detection [54]. The use of the Internet and social networks is not exempt of risks, a concerning rise on misinformation has been reported leading to confusion and risking the patient’s health; however, this variable was not studied in the present research [55].

## Conclusions

In conclusion, a positive temporal correlation was observed between the interest on breast cancer and awareness campaigns; this temporal correlation was lower in prostate cancer but showed a higher median RSV. Significant variation was found in the interest in the neoplasms evaluated in the period of study, with significant increases in all except ‘cervical cancer’. Despite the fact that cervical cancer is one of the main neoplasms in Peru, the little interest of the population is striking because it would be important to stratify the campaigns aimed at this neoplasm. Cancer incidence in certain regions was geographically related to higher RSV as in the case of breast, cervical and colorectal cancer. Monitoring the RSV using Google Trends could help to identify populations’ interests in cancer screening and may complement traditional data collection about public health awareness programmes.

## Authors’ contributions

Jorge Luna-Abanto conceived of the idea of an article on this topic and developed it. Jorge Luna-Abanto and Luis Gamarra wrote the article and created the graphics. All authors discussed the review and contributed to the final manuscript.

## Ethics approval and consent to participate

Not applicable.

## Consent for publication

Not applicable.

## Data availability

All data generated or analysed during this study are included in this published article and its supplementary information files.

## Funding information

The authors received no specific funding for this work.

## Conflicts of interest

None.

## Figures and Tables

**Supplementary Table S1.** Population aged 6 and over using the Internet, according to language and geographic area, Peru: 2016–2020.

	(Percentage)				
Language/geographic area	2016	2017	2018	2019	2020
Total	**45.5**	**48.7**	**52.6**	**57.2**	**64.9**
Spanish	51.4	55.0	58.9	63.2	70.3
Other native language	16.1	17.7	20.0	24.9	37.2
Geographic area					
Urban	**54.6**	**58.2**	**62.2**	**66.4**	**71.9**
Spanish	58.6	62.3	66.1	70.1	75.5
Other native language	22.7	26.2	29.5	35.2	44.8
Rural	**14.2**	**15.4**	**17.7**	**22.6**	**36.7**
Spanish	17.0	19.3	22.2	27.6	42.0
Other native language	9.4	8.8	10.0	13.6	26.6
Geographic area					
Coast	**57.6**	**62.0**	**66.3**	**70.9**	**76.3**
Spanish	60.0	64.4	68.5	72.8	78.1
Other native language	23.7	28.6	31.9	38.1	50.3
Highland	**31.4**	**32.5**	**36.1**	**40.3**	**50.9**
Spanish	40.6	42.0	46.0	49.7	60.8
Other native language	15.1	15.8	18.4	23.1	34.2
Jungle	**27.5**	**30.7**	**33.0**	**38.3**	**47.6**
Spanish	30.8	34.6	36.8	42.3	49.9
Other native language	7.2	7.9	9.2	12.4	23.0

^a^Includes the population whose mother language is Quechua, Aymara and Amazon

^b^Includes the Province of Lima and the Constitutional Province of Callao

Source: Instituto Nacional de Estadística e Informática - Encuesta Nacional de Hogares
